# Changes in Medical Faculty

**Published:** 1853-08

**Authors:** 


### CHANGES IN THE FACULTY.

Some changes have occurred in the Faculty of the Medical College, during the past few months, which we briefly announce.

The Chair of Surgery having been vacated in February last, a short time previous to the close of the last course of lectures, the place was filled by the appointment of Dr. Hughes, formerly Prof. of Anatomy.

Dr. McGugin having been transferred to the Chair of Physiology and Pathology,

E. R. Ford, M. D. of Keokuk, was appointed to the Chair of Obstetrics, vacated by Dr. McGugin. Dr. F. has been seven years a successful practitioner in this city, and is extensively and favorably known to the medical community.

Edward A. Arnold, M. D. of Baltimore, has been appointed to the Chair of Anatomy, vacated by Dr. Hughes. Dr. Arnold holds an eminent position in his profession; he was for three years Physician in one of the public Hospitals of Baltimore, and has been a careful and thorough student of his profession, as a science. We are pleased to be able to announce that he has located permanently at Davenport, for we esteem the accession of such men to the body of Western practitioners, an omen of good for the position and progress of the medical profession, in this portion of the country.

